# Satellite data for Singapore, Manila and Kuala Lumpur city growth analysis

**DOI:** 10.1016/j.dib.2016.04.028

**Published:** 2016-04-22

**Authors:** Mukesh Singh Boori, Komal Choudhary, Alexander Kupriyanov, Viktor Kovelskiy

**Affiliations:** a Samara National Research University, Samara, Russia; b American Sentinel University, CO, USA; c Image Processing Systems Institute, Samara, Russia; d Hokkaido University, Sapporo, Japan

**Keywords:** Urban growth, City density, Landsat satellite data, Change detection, Remote sensing, GIS, Singapore, Manila, Kuala Lumpur

## Abstract

This data article presents satellite data related to city growth of Singapore, Manila and Kuala Lumpur cities. The data were collected from NASA and USGS websites. A method has been developed for city built-up density from city center to outward till 50 km by using satellite data. These data sets consists three decade Landsat images. A detailed description is given to show how to use this data to produce urban growth maps. The urban growth maps have been used to know the changes and growth pattern in the Southeast Asia Cities.

## **Specifications Table**

**Table:** 

Subject area	Earth Science and Geo-informatics
More specific subject area	Remote Sensing and GIS
Type of data	Satellite image, table, figure, graph
How data was acquired	Collect from field and download from NASA and USGS website
Data format	Analyzed
Experimental factors	Image processing
Experimental features	Image classification, combined satellite data and socio-economic data in GIS with the help of ArcGIS 10.2 software
Data source location	Scientific Research Laboratory of Automated Systems of Scientific Research (SRL-35), Samara State Aerospace University, Samara, Russia
Data accessibility	Data is in this data article

## Value of the data

• City growth and land use/cover data are utilize in maximum type of remote sensing data applications such as hydrology, agriculture, forest, urban growth/city planning, vulnerability, natural resources and sustainable development etc.
• Socio-economic or secondary data such a general amenities, facilities and field data are useful to verify the satellite data and to know the changes of an area.
• Data of urban expansion, land use/cover are very useful to local government and urban planners for the future plans to sustainable development of the city.

## 1 Data

**Fig. 1:**
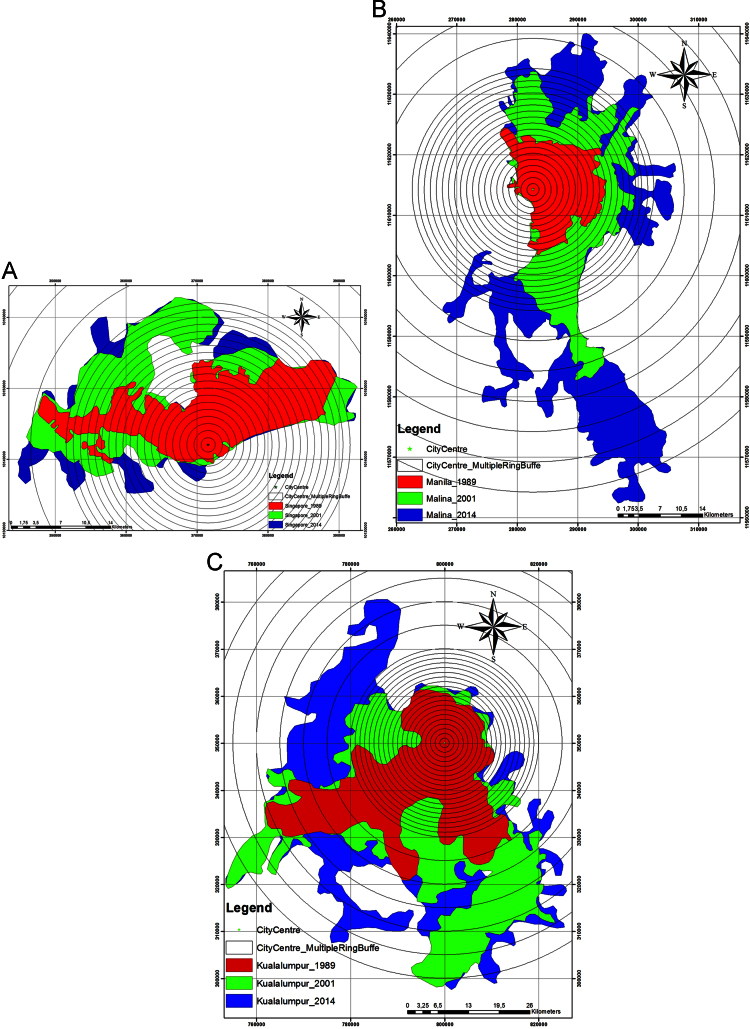
Singapore, Manila and Kuala Lumpur city growth from 1989, 2001 and 2014.

The following multi-sensor, multi-resolution and multi-temporal satellite data were used: Landsat 5 TM (Thematic Mapper) for 1989, Landsat 7 ETM+ (Enhanced Thematic Mapper plus) for 2001 and Landsat 8 OLI (Operational Land Imager) for 2014, an image captured by a different type of sensors with less than 20% cloud cover. In addition, Landsat images are frequently updated and are available free of charge through the Global Land Cover Facility repository [1], [2], [3]. All data were downloaded free of charge from NASA and USGS website. Fig. 1 presents city growth of Singapore, Manila and Kuala Lumpur in the last three decades.

## 2 Experimental design, materials and methods

**Fig. 2:**
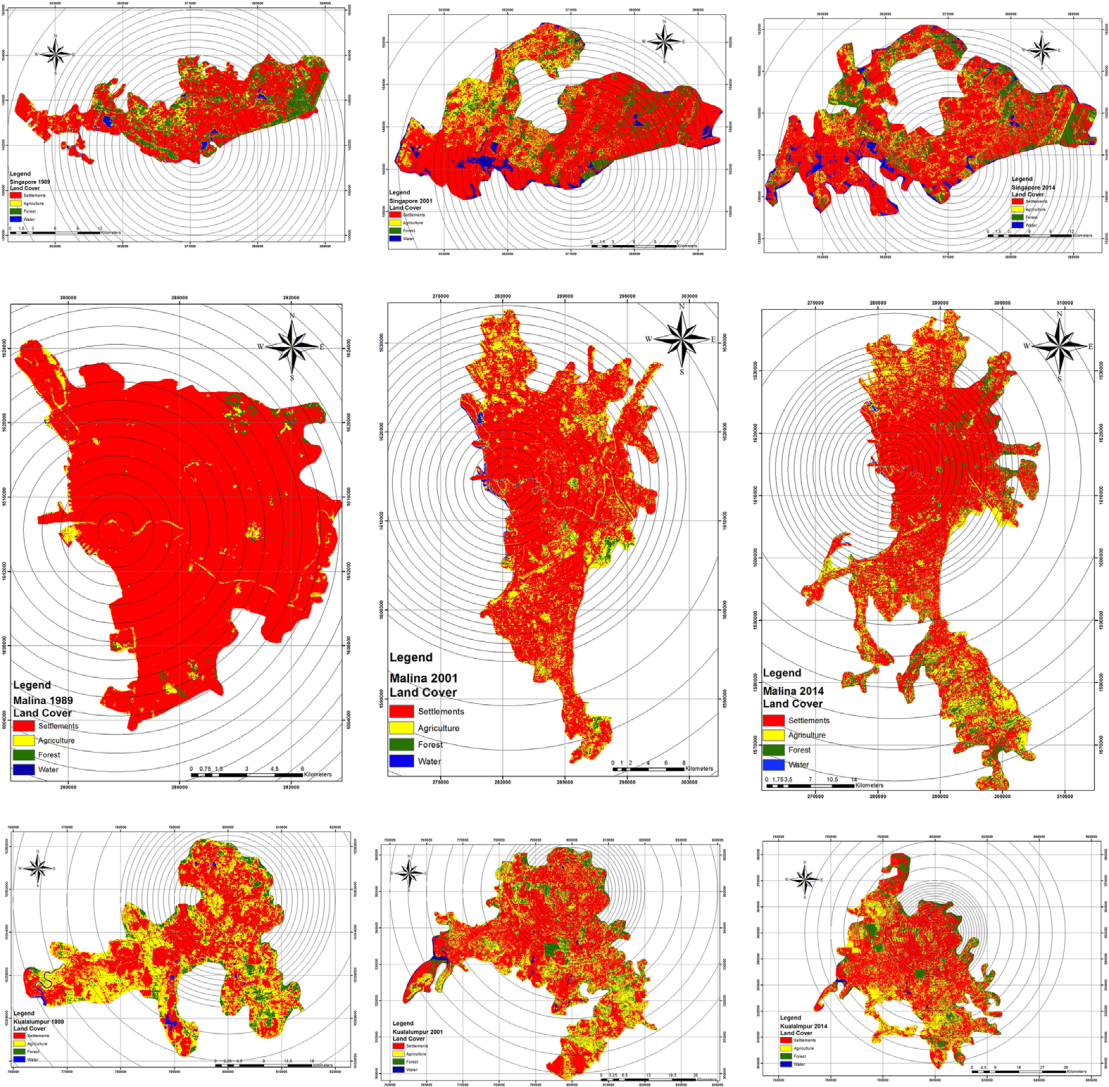
Land cover and multi-buffer ring zones around city center of Singapore, Manila and Kuala Lumpur for the year of 1989, 2001 and 2014.

To convert all row satellite data into meaningful data, first rectified and georeferenced the data in UTM projection (WGS 84 datum). To increase the quality of satellite data, all data processed through band ratio, classification and in last change detection techniques [4], [5], [6]. All satellite data were preferred to retain the spatial details such as original pixel value and size. Therefore the satellite data were kept without changing their pixel sizes despite the possible varying accuracy level of classification with the different spatial, spectral and radiometric resolutions. All nine satellite images were classified through maximum likelihood supervised classification in ArcGIS 10.2 software [7], [8], [9]. To create a closer correspondence between the produced output data maps, the classification was done by only considering four main classes: urban/built-up area, agriculture land, forest land (tree/park) and water body [10], [11], [12]. The produced data maps were presented in Fig. 2.

After classification multi-buffer rings were created for every 1 km distance from 1 to 50 km from city center to outward. Than intersect with classified data maps (land cover) for all three dates [13], [14], [15]. Later on all land cover classes area were measured from 1 to 50 km distance and derive urban density according to following formula.

(1) $$\mathrm{Urban density}=\frac{\mathrm{Sattlement}\;\mathrm{area}/\mathrm{ring}}{\mathrm{Total}\;\mathrm{ring}\;\mathrm{area}}$$

In all buffer rings only urban/built-up area was calculated in place of whole ring area. After producing complete land use data maps, the total coverage of different classes were determined. Using this information, we calculated the water, forest, vegetation and built-up area per capita for all the study years (Table 1).

In order to evaluate the spatial distribution of urban expansion intensity, we adapted an indicator called annual urban growth rate (AGR) for evaluating the ‘urbanization’ speed of per unit area [16], [17]. AGR is defined as follows:

(2) $$\mathrm{AGR}=\frac{{\mathrm{UA}}_{n+i}-{\mathrm{UA}}_{i}}{{\mathrm{nTA}}_{n+i}}\times 100\%$$

where *TA*_*n*+*i*_ is the total land area of the target unit to be calculated at the time point of *i*+*n*; *UA*_*n*+*i*_ and *UA*_*i*_ the urban area or built-up area in the target unit at time *i*+*n* and *i*, respectively and *n* is the interval of the calculated period (in years).

### 2.1 Urban/built-up area, city density and landscape

**Fig. 3:**
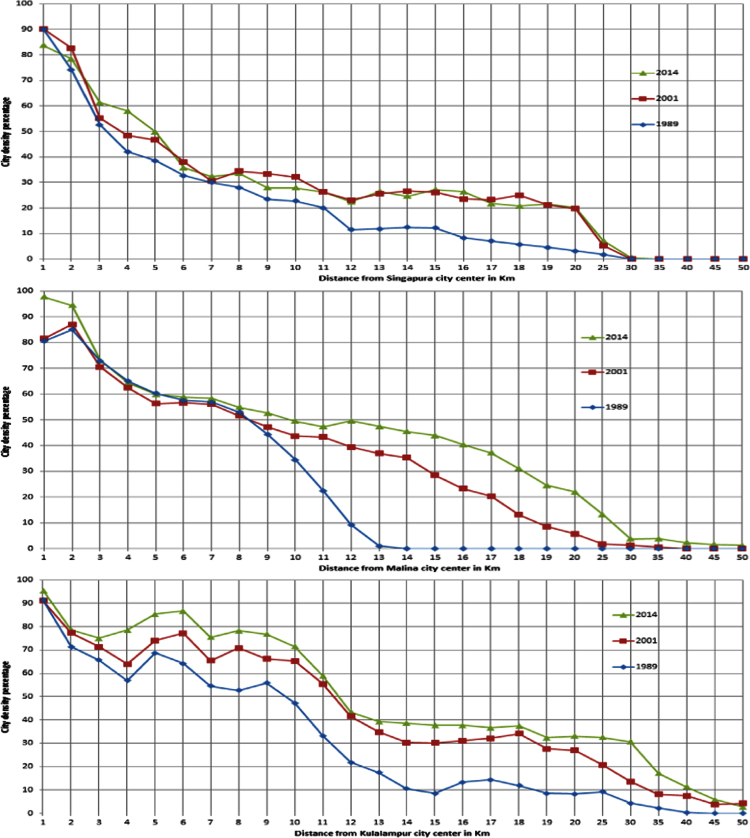
Singapore, Manila and Kuala Lumpur city density from 1 to 50 km distance for the year of 1989, 2001 and 2014.

With the help of ArcGIS 10.2 software, urban/built up area data were created. After creation of built-up data, city density data were generated by Eq. (1) from city center to 50 km outward the city for Singapore, Manila and Kuala Lumpur cities (Fig. 3).

As Fig. 3 represents three dates data of urban density so this data can be utilize as urban growth rate data of Singapore, Manila and Kuala Lumpur city.

**Fig. 4:**
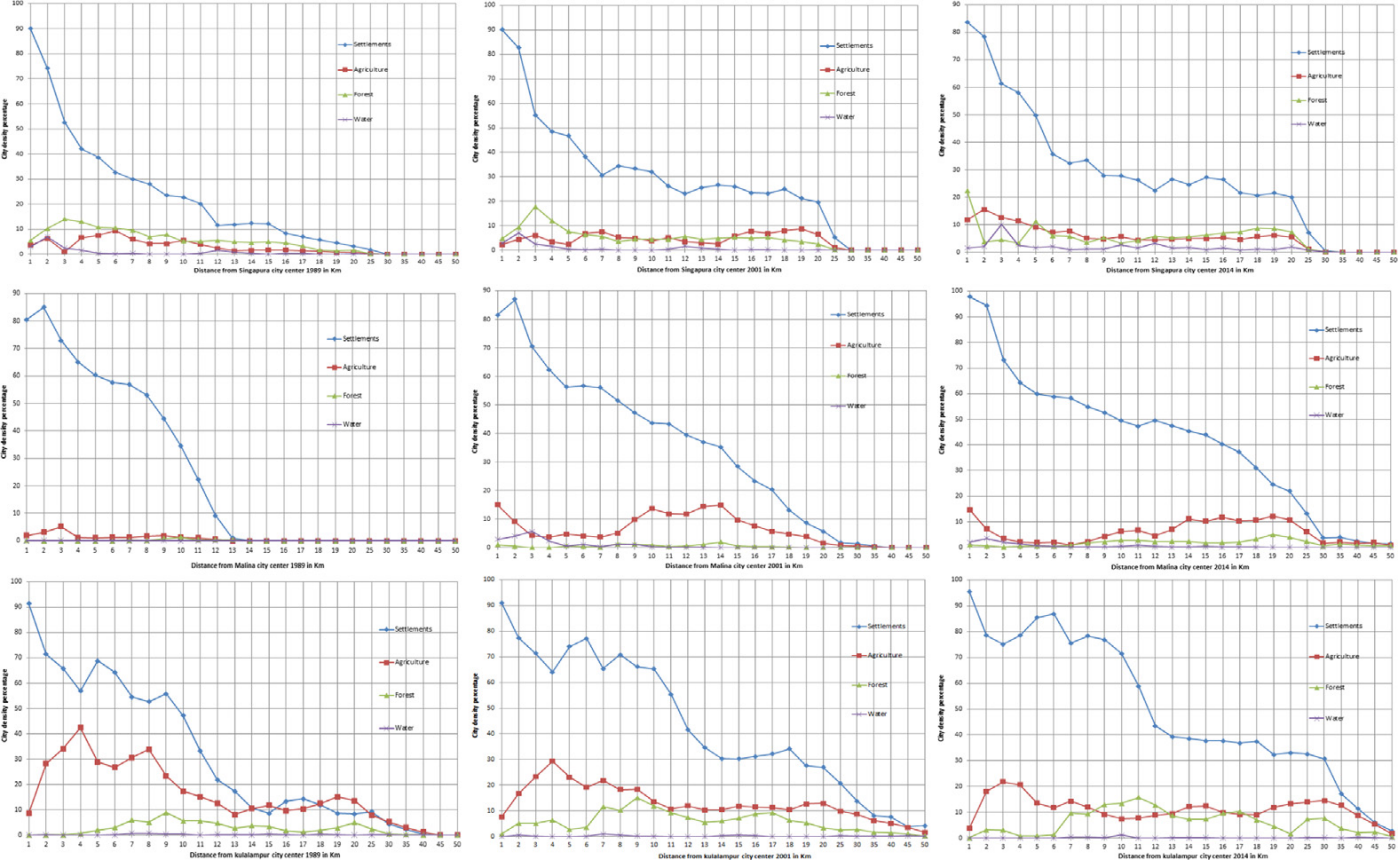
Singapore, Manila and Kuala Lumpur land cover class density from 1 to 50 km distance for the year of 1989, 2001 and 2014.

With the help of supervised classification, previous knowledge and experience, landscape data were created for all three cities for the year of 1989, 2001 and 2014. Here a trimble hand-held GPS with an accuracy of 10 m was used to map and collect the coordinates of important landscape features during pre- and post-classification field visits in order to prepare land-use and land-cover datasets. Fig. 4 shows landscape data with urban growth and urban density datasets for Singapore, Manila and Kuala Lumpur cities from 1989 to 2014.

## Figures and Tables

**Table 1:** Singapore, Manila and Kuala Lumpur land use/cover classes for 1989, 2001 and 2014.

	**Area_1989**	**%**	**Area_2001**	**%**	**Area_2014**	**%**
**Singapore**						
Settlements	196.88	66.25	383.23	69.71	464.69	70.19
Agriculture	34.67	11.67	80.26	14.60	80.84	12.21
Forest	60.74	20.44	62.58	11.38	89.65	13.54
Water	4.89	1.65	23.64	4.30	26.87	4.06
Total	297.18	100.00	549.71	100.00	662.05	100.00
						
**Manila**						
Settlements	189.61	96.70	416.87	77.08	776.65	69.12
Agriculture	6.37	3.24	110.14	20.37	243.89	21.71
Forest	2.19	1.11	9.44	1.75	98.59	8.77
Water	0.1	0.05	4.36	0.81	4.42	0.39
Total	196.08	100.00	540.81	100.00	1123.55	100.00
						
**Kuala Lumpur**						
Settlements	456.99	51.81	1098.48	60.22	1663.23	64.36
Agriculture	345.46	39.16	520.40	28.53	699.21	27.06
Forest	70.50	7.99	188.74	10.35	209.99	8.13
Water	9.18	1.04	16.54	0.91	11.68	0.45
Total	882.13	100.00	1824.16	100.00	2584.11	100.00
